# Viral highway to nucleus exposed by image correlation analyses

**DOI:** 10.1038/s41598-018-19582-w

**Published:** 2018-01-18

**Authors:** Elina Mäntylä, Jenu V. Chacko, Vesa Aho, Colin R. Parrish, Victor Shahin, Michael Kann, Michelle A. Digman, Enrico Gratton, Maija Vihinen-Ranta

**Affiliations:** 1 Department of Biological and Environmental Science and Nanoscience Center, University of Jyvaskyla, Jyvaskyla, Finland; 2 Laboratory for Fluorescence Dynamics, University of California, Irvine, California, USA; 3 Department of Biomedical Engineering, University of California, Irvine, California, USA; 4 Department of Physics, and Nanoscience Center, University of Jyvaskyla, Jyvaskyla, FI-40500 Finland; 5 Baker Institute for Animal Health, College of Veterinary Medicine, University of Cornell, Ithaca, USA; 6 Institute of Physiology II, University of Münster, Münster, Germany; 7 University of Bordeaux, Microbiologie Fondamentale et Pathogénicité, Bordeaux, France

## Abstract

Parvoviral genome translocation from the plasma membrane into the nucleus is a coordinated multistep process mediated by capsid proteins. We used fast confocal microscopy line scan imaging combined with image correlation methods including auto-, pair- and cross-correlation, and number and brightness analysis, to study the parvovirus entry pathway at the single-particle level in living cells. Our results show that the endosome*-*associated movement of virus particles fluctuates from fast to slow. Fast transit of single cytoplasmic capsids to the nuclear envelope is followed by slow movement of capsids and fast diffusion of capsid fragments in the nucleoplasm. The unique combination of image analyses allowed us to follow the fate of intracellular single virus particles and their interactions with importin β revealing previously unknown dynamics of the entry pathway.

## Introduction

Recent studies elucidating the dynamics of nuclear-replicating virus entry into host cells have shown that the capsid transport from the plasma membrane into the nucleus consists of mechanistically distinct dynamic stages^1–6^. These involve both directed movement and diffusion in the molecularly crowded environment of the cell interior. These movements depend on the physical properties of the capsids such as their size and interactions with intracellular factors^1,3,7–9^. Viral capsid movement is further affected by their time- and location-dependent concentration as well as the clustering of capsids. This complexity poses a major challenge for fluorescence microscopy-based determination of viral entry dynamics in particular for quantitative analysis with single particle precision^10–14^.

**Figure 1:**
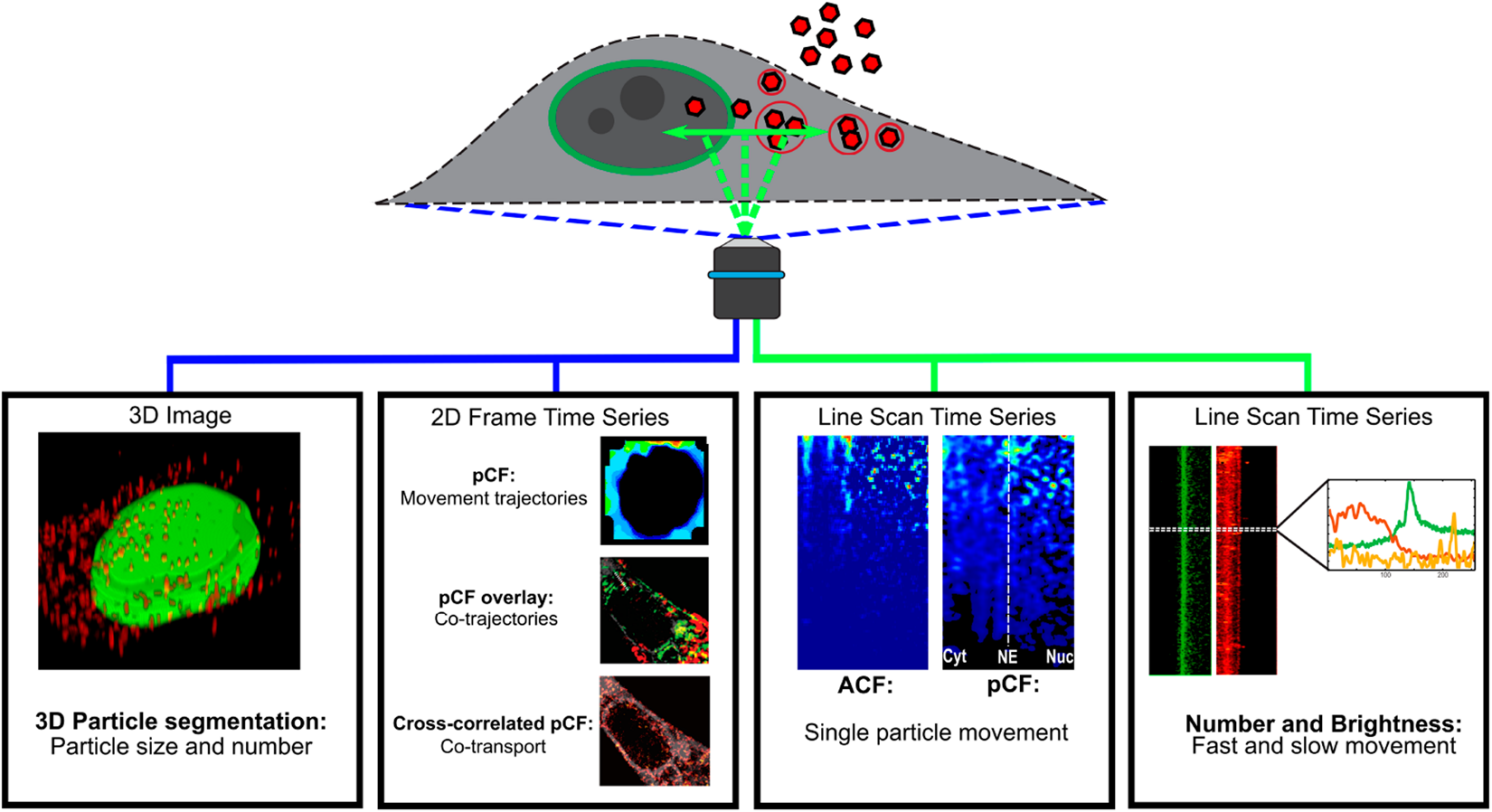
Overview of image acquisition and analyses workflow. The laser scanning confocal microscopy (LSCM) line scans of viral capsids were studied by 3D particle segmentation analysis, and fluorescence correlation spectroscopy -based methods such as auto- and pair correlation (pCF) function, and number and brightness (N&B) analyses. The motion and interaction of capsids were investigated by two-color pCF overlay and pCF cross-correlation analyses.

Here, we employed 3D particle segmentation analyses of laser scanning confocal microscopy (LSCM) images to quantify the distribution and motility of viral particles. Number and brightness (N&B) analysis^15^ and fluorescence correlation spectroscopy (FCS) -derived advanced image analyses along with confocal line scans were used to quantify the dynamics of capsid movement in living cells on their way towards the nucleus^16^. FCS is a tool for studying particle dynamics using their fluorescence intensity fluctuations. Here, we used the FCS parameters from LSCM to quantify capsid dynamics by autocorrelation function (ACF) and pair correlation function (pCF) of the fluorescence fluctuations and brightness analysis of the molecules over time. In addition, cross-correlated pair correlation image analysis was used to visualize the correlated transport of capsids across the nuclear envelope (NE) (Fig. 1). Correlation functions enable the calculation of viral population behavior within a given region of the cell with a high temporal resolution (microsecond scale) and spatial resolution limited only by the speed of scanning and diffraction^17–19^. N&B analysis, measuring the average number of molecules and their brightness in each pixel^15^, allowed the quantitative assessment of the number of fluctuating viral capsids in the entire cell. Finally, cross-correlated image pCF of the capsids and importin β revealed their simultaneous transport across the NE independently of the brightness and total number of particles^16^. Together, these techniques allowed a precise analysis and visualization of capsid transport across the NE thereby permitting a more complete description of single capsid dynamics.

Parvoviruses, small DNA viruses with high potential as gene delivery vectors and oncolytic agents in cancer therapeutics, enter cells via clathrin-mediated endocytosis^20–26^. Endosomal acidification-induced capsid release into the cytosol is followed by dynein- and microtubule-dependent transport of cytoplasmic capsids towards the nucleus^5,24,27–37^. The relatively limited number of cytoplasmic capsids makes it difficult to distinguish them from intravesicular capsids targeted to lysosomal degradation. Parvoviral capsids (~26 nm in diameter) enter the nucleus due to their dependence on the cellular DNA replication machinery. Classically, the transport of macromolecules across the NE is mediated by transport receptors of the importin β superfamily^38^. Importin β interacts either directly with cargo exposing an importin β binding domain, or indirectly via importin α bound to cargo possessing nuclear localization signals^39^. After nuclear import, the importin β-cargo complex is dissociated at the nuclear side of the nuclear pore complex (NPC). Although parvovirus capsids contain classical nuclear localization signals^40^, it is currently unknown whether importins are involved in the nuclear import of parvoviruses. Nuclear admission of the viral capsids is followed by a so far unknown process of viral genome release into the nucleoplasm. Due to their small size, the imaging of parvovirus entry by conventional microscopy techniques is challenging, particularly in real time in living cells on the single capsid level. This study combined advanced fluorescence correlation analyses, which allowed for a comprehensive description of parvoviral single capsid dynamics at different locations in the cell and allowed correlating these findings with distinct mechanistic events during capsid transport. In particular, our results describe the varied intracellular kinetics of capsid entry, and the discovery of capsid interaction with importin β during their nuclear import, increasing our comprehensive understanding of the parvoviral nuclear entry pathway.

## Results

### Cytoplasmic distribution of capsids

**Figure 2:**
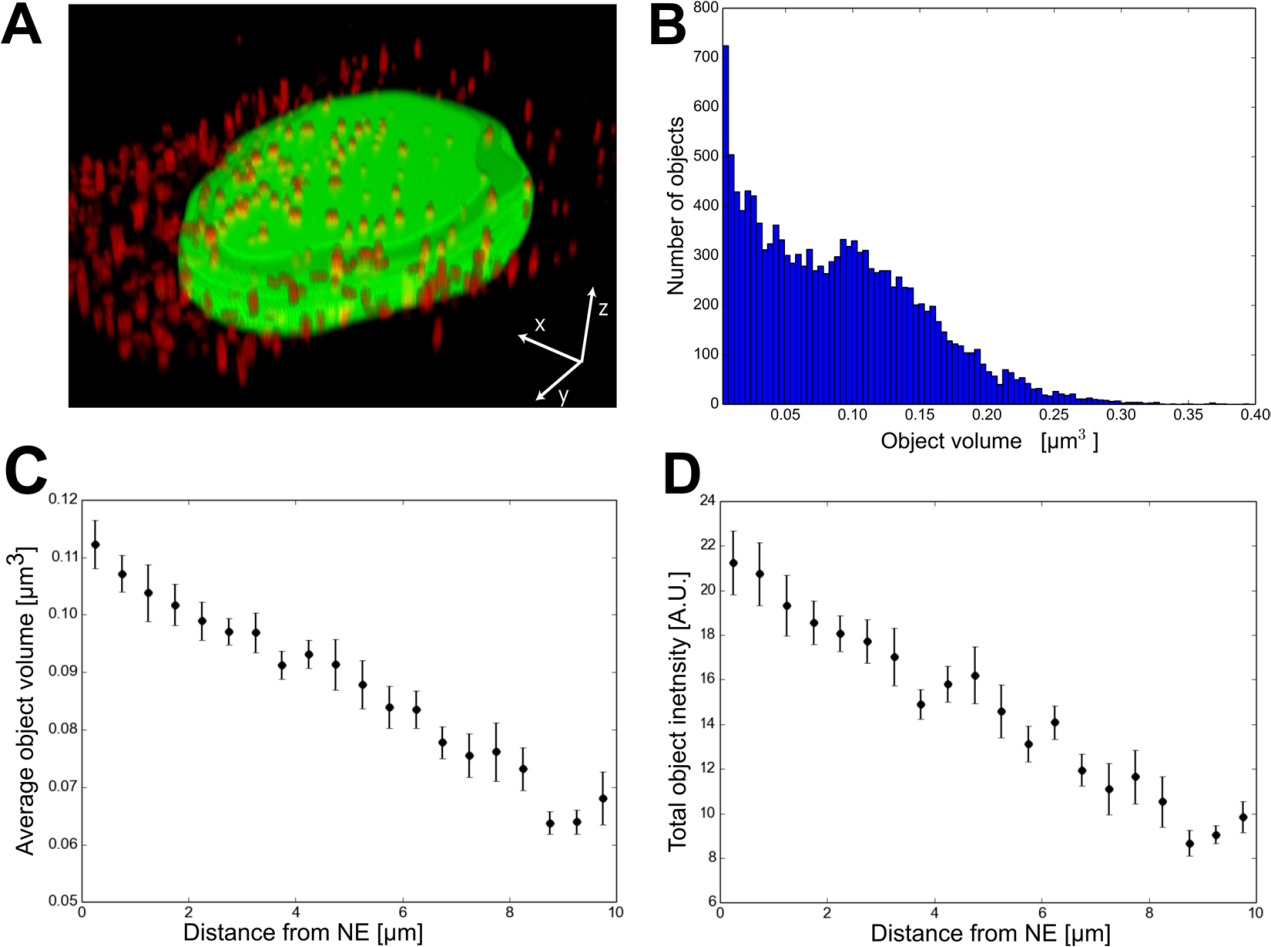
Intracellular capsid distribution. (**A**) 3D reconstruction of a confocal image showing distribution of A594 labeled capsids (red) and lamin C-EGFP (green) at ~1 h p.i. (**B**) 3D segmentation particle analysis of number of virus-containing cytoplasmic particles as a function of their volume, (**C**) an average volume and (**D**) total intensity as a function of the distance from the NE. The black dots represent the means and the error bars represent the ± standard error of mean (±SEM).

To follow viral trafficking towards the nucleus, we analysed the cytoplasmic distribution of canine parvovirus (CPV) capsids as a prototype for other parvoviruses. Live-cell confocal imaging showed viral particles in distinct spherical cytoplasmic structures at 1 h post infection (p.i.) (Fig. 2A). 3D particle segmentation analysis (n = 20 cells) revealed that the capsid-containing structures showed a broad distribution in size (Fig. 2A and B). It also disclosed simultaneous accumulation of not only the largest but also the brightest structures in the nuclear periphery (Fig. 2C and D). The detected increase in structure volume positively correlated with an increase in structure intensity. This suggests that the larger objects result from the fusion of capsid-containing vesicles amongst each other rather than with non-capsid vesicles. Perinuclear accumulation of capsids in the cytoplasm was accompanied by the presence of smaller, less bright structures in the cell periphery. As parvoviruses enter the cell via the endosomal system, it is plausible that the large structures correspond to endosomes loaded with multiple capsids. Considering that single capsids should have weaker fluorescence, visualization of these particles entails higher detection sensitivity.

### Intracellular dynamics of viral capsids

**Figure 3:**
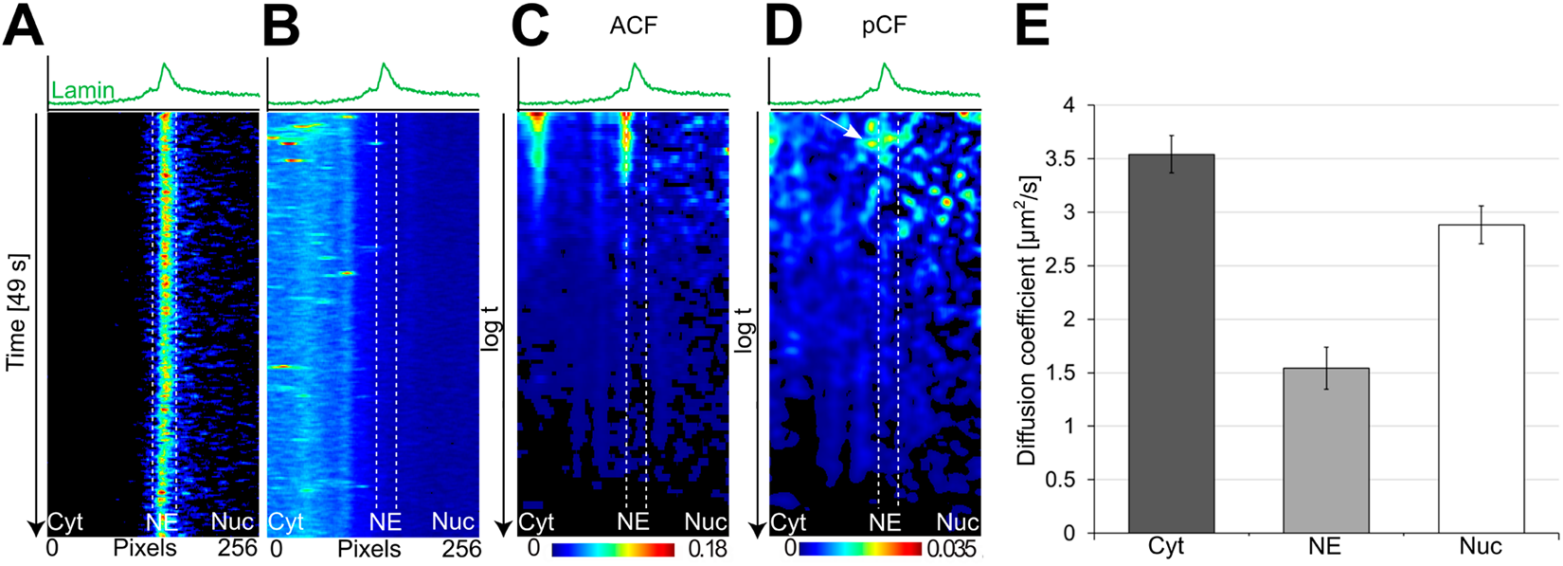
Image correlation analysis of capsid movement. (**A**) Average fluorescence intensity image showing the position and fluctuations of lamin C-EGFP. (**B**) Cytoplasmic capsid intensity trajectories along the scanned line in the cytoplasm, at the nuclear envelope (NE) and in the nucleoplasm as a function of time and distance are shown. (**C**) Autocorrelation function (ACF) analysis of intracellular viral movement. (**D**) Pair correlation function (pCF) analysis showing a positive correlation across the lamin C-EGFP and viral capsid transit over NE (arrowhead). Pseudocolor coloring with intensity increasing from blue to red demonstrates the distribution of fluorescence intensity. X axis represents distance in pixels along the line scan and y axis is logarithmic time scale. (**E**) File averaged diffusion coefficients from the ACF analysis of capsid movement in the cytoplasm, at the NE and in the nucleoplasm (38 measurements from 14 cells) are shown as boxplots. The error bars represent the ± standard error of mean (±SEM).

For monitoring cytoplasmic dynamics and nuclear import of capsids, their dynamics were characterized using two-channel acquisition of line scans (n = 15) in lamin C-EGFP-expressing cells infected for 40–110 min with A594-labeled CPV capsids. The average fluorescence intensities of EGFP and A594 from 32,000 scanned lines were plotted to identify the position of lamina, the distribution of viral particles, and the change in their intensity over the imaging time (Fig. 3A and B). Consistent with the 2D/3D particle distribution analyses (Fig. 2), the intensity maps demonstrated cytoplasmic dynamics and accumulation of particles in close proximity to the nuclear rim (Fig. 3B). Based on their distance from the nuclear lamina the pixels along the line scans were categorized into three distinct zones of particle dynamics: the cytoplasm, the NE and the nucleoplasm. The cytoplasm (zone 1) was dominated by frequent particle movement. The NE (zone 2, width of ~22 pixels corresponding to ~440 nm around the nuclear lamina) was characterized by low-frequency particle movement (zone 2). This intensity analysis could not detect any particle movement in the nucleoplasmic zone (zone 3). These results suggest that CPV-infected cells have several spatio-temporally distinct zones of capsid dynamics, with the motility regulated by putative changes in microviscosity and/or cellular infrastructure.

The fluorescence fluctuations of the viral capsids close to the NE were characterized using ACF across the nuclear lamina over time. The ACF correlates the fluorescence signal fluctuating in time with itself in each pixel. The ACF revealed the presence of dynamic particles in the cytoplasm and in particular in close proximity to the NE (Fig. 3C). In line with our particle distribution analyses (Fig. 2) the presence of very slowly moving (nearly immobile) particles near the NE was observed. ACF significantly improved the temporal resolution of our analyses from millisecond to microsecond scale. However, this approach, limited by a low number of observations of the particle fluctuations, failed to identify potential single capsid dynamics in the NE and the nucleoplasm.

To follow single capsid movement across the NE, we used pCF, which plots the correlation of spatially separated pixels, enhancing the correlation of single particle dynamics between those two points in space. First, we captured the transit of single particles across the NE by correlating and plotting two spatially separated pixels ~800 nm (20 pixels) apart (Fig. 3D). A positive correlation (curved trajectory) signal on the pair correlation carpet across the lamin C signal verified that the capsids were transported from the cytoplasm across the NE into the nucleoplasm (Fig. 3D). These results indicated that the pCF approach enabled the detection of rare single capsid dynamics and revealed a complete viral nuclear import event over the NE. The presence of capsids inside the nucleus was confirmed in a separate assay by atomic force microscopy (AFM) (Supplementary Fig. S1). We then used mathematical fitting of the ACF to determine the diffusion coefficients (*D*) of particles in the cytoplasm, at the NE and in the nucleoplasm (Fig. 3E). The goodness-of-fit was validated using chi-square test and the analysis was limited to a diffusion range of 0–100 µm^2^/s. For plotting, the data were spatially categorized into the three distinct zones as before. The analysis of the average diffusion of all particles showed that, in comparison to the cytoplasm (mean D ± standard error of the mean, *D* = 3.53 ± 0.17 µm^2^/s) the movement of particles was significantly slowed down at the NE (*D* = 1.54 ± 0.20 µm^2^/s) (Fig. 3E). Analyses of nucleoplasm showed the presence of multiple peaks of diffusion coefficient for the viral particles. The ACF fitting allowed categorization of the particle movement into fast (*D* > 2 µm^2^/s) and slow movement (*D* ≈ 0–2 µm^2^/s). Accordingly, the observed particle diffusion in the nucleus was 2.88 ± 0.18 µm^2^/s with an average of *D* ≈ 1 µm^2^/s for the fast and *D* = 0.35 µm^2^/s for the slow (Fig. 3E; see also Supplementary Fig. S2). This could indicate the presence of intact capsids but also their rapidly moving fragments and detached dye molecules, arguing for intranuclear capsid disassembly.

### Quantification of capsid nuclear entry

To specify the dynamics of the capsids in the cytoplasmic, NE and nucleoplasmic zones, quantitative LSCM line scan -based N&B analysis was performed (on 38 line scan images, n = 15). This line scan approach allows the detection of very rapid fluorescence fluctuations enabling the description of the dynamics of single fluorescent viral particles. Moreover, N&B provides an intensity-independent brightness/particle -ratio which was used here as a quantitative measure of the relative particle content of an endosome.

**Figure 4:**
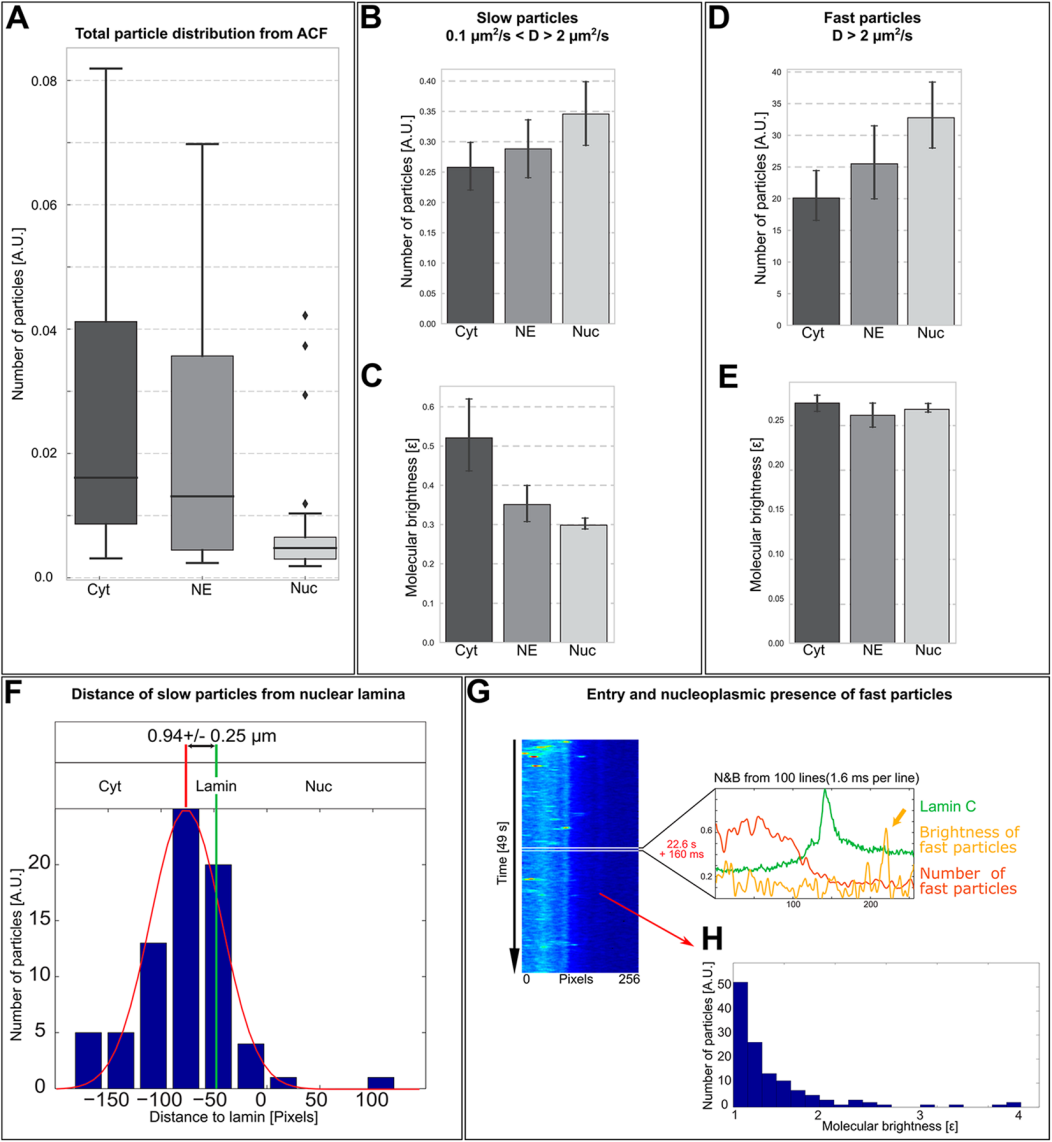
Number and brightness analysis of capsid dynamics. (**A**) ACF based file averaged analysis showing the number of all moving particles in the cytoplasm (dark gray), at the NE (light gray) and in the nucleus (white) (n = 15 cells). Number (**B**,**C**) and brightness (**D**,**E**) analyses of fast and slow moving particles in the cytoplasm, NE and nucleus. The black horizontal lines represent the median, the crosses represent the mean, and boxes represent the quartiles of data range including the median. (**F**) A histogram of the average brightness of the slow fluorescent viral particles as a function of distance from the nuclear lamina in all cells studied. (**G**) Single-cell fluorescence intensity map showing the number (red) and brightness (yellow) of cytoplasmic and nuclear capsids and distribution of lamin C (green, time-average of 100 line scans). (**H**) Histogram showing absolute brightness distribution of fast nuclear viral particles.

N&B analysis of particles categorized as fast (*D* > 2 µm^2^/s) and slow (*D *≈ 0.1–2 µm^2^/s). The number distribution demonstrated that the majority of particles (79%) were distributed in the cytoplasm and the NE area, with only a smaller fraction of them located in the nucleoplasm (21%) (Fig. 4A). The number analysis showed that the cytoplasm and the NE area contained both fast (20.1 ± 1.82; 25.5 ± 2.38) and slow (0.26 ± 0.02; 0.29 ± 0.02) particles (Fig. 4B and C; see also Supplementary Table S1). The observation of fast particles in the cytoplasm and the NE area suggest the presence of capsids in fast moving endosomes and/or cytoplasmic capsids released from the endosomes. The presence of slow moving viral particles in the cytoplasm possibly within large endosomes was also detected, but the accumulation of particles in large immobile endosomal vesicles was not taken into account in this motion-based analysis. In the nucleoplasm, the numbers of fast and slow particles were 32.99 ± 2.68 and 0.35 ± 0.03, respectively (Fig. 4B and C; see also Supplementary Table S1). This suggests that the intranuclear motility of capsids includes both fast diffusion of capsid remnants and/or detached fluorophores in addition to slow moving capsids possibly retained by the nuclear baskets of NPCs or other nuclear structures.

The brightness analysis indicated that both the fast and slow particles were present in the cytoplasm (0.27 ± 0.01; 0.52 ± 0.05) and in the NE area (0.26 ± 0.01; 0.35 ± 0.02; Fig. 4D and E; see also Supplementary Table S1). The finding of fast and bright cytoplasmic particles is consistent with our image correlation and number analyses, which showed the presence of cytoplasmic fast-moving virus*-*containing endosomes or cytoplasmic viruses. The observation of cytoplasmic slow or nearly immobile bright particles is in line with 2D/3D particle analysis (Fig. 2) showing capsid accumulation into large perinuclear endosomal vesicles. The brightness of the slow particles, depending on the amount of fluorescent capsids inside them, was highest in the cytoplasm at 0.94 ± 0.25 µm away from the lamina (Fig. 4F). This could describe immobilization of virus-containing vesicles, since large and very slow or immobile particles aggregates generally appear much brighter than small, fluctuating molecules or even small aggregates in this analysis method. Notably, at the nuclear periphery only fast particles were observed to translocate to the NE suggesting that single cytosolic capsids actively move toward the nucleus after their release from endosomes (Fig. 4G; see also Supplementary Table S1). In line with our pCF analysis (Fig. 3D), fast and slow nucleoplasmic particles with brightness of 0.35 and 32.9, respectively, were detected. Importantly, brightness of the fast particles in the cytoplasm, at the NE and in the nucleoplasm was nearly equal to that of the nucleoplasmic slow particles pointing to translocation of capsids from the cytoplasm into the nucleus (Fig. 4D and E). Finally, the nuclear entry of capsids was rare as indicated by the low number (Fig. 4A) and infrequent presence of intranuclear slow particles (Fig. 4E). The detection efficacy of intranuclear infrequent particles was enhanced by a peak detection algorithm (Fig. 4H). This analysis showed that the presence of intranuclear bright viral particles was low, which suggests that the nuclear import of capsids is a rare event.

Altogether, quantitative N&B analysis revealed that the majority of capsids accumulated in slow perinuclear vesicles. This was accompanied by the presence of fast-moving and equally bright particles corresponding to single capsids both in the NE border area and within the nucleus. However, the analysis showed the presence of slow capsids at the NE and in the nucleus, which indicated that capsid movement was slowed down by the NE, NPC or chromatin.

### Capsid interactions with importin β after cytosolic release

**Figure 5:**
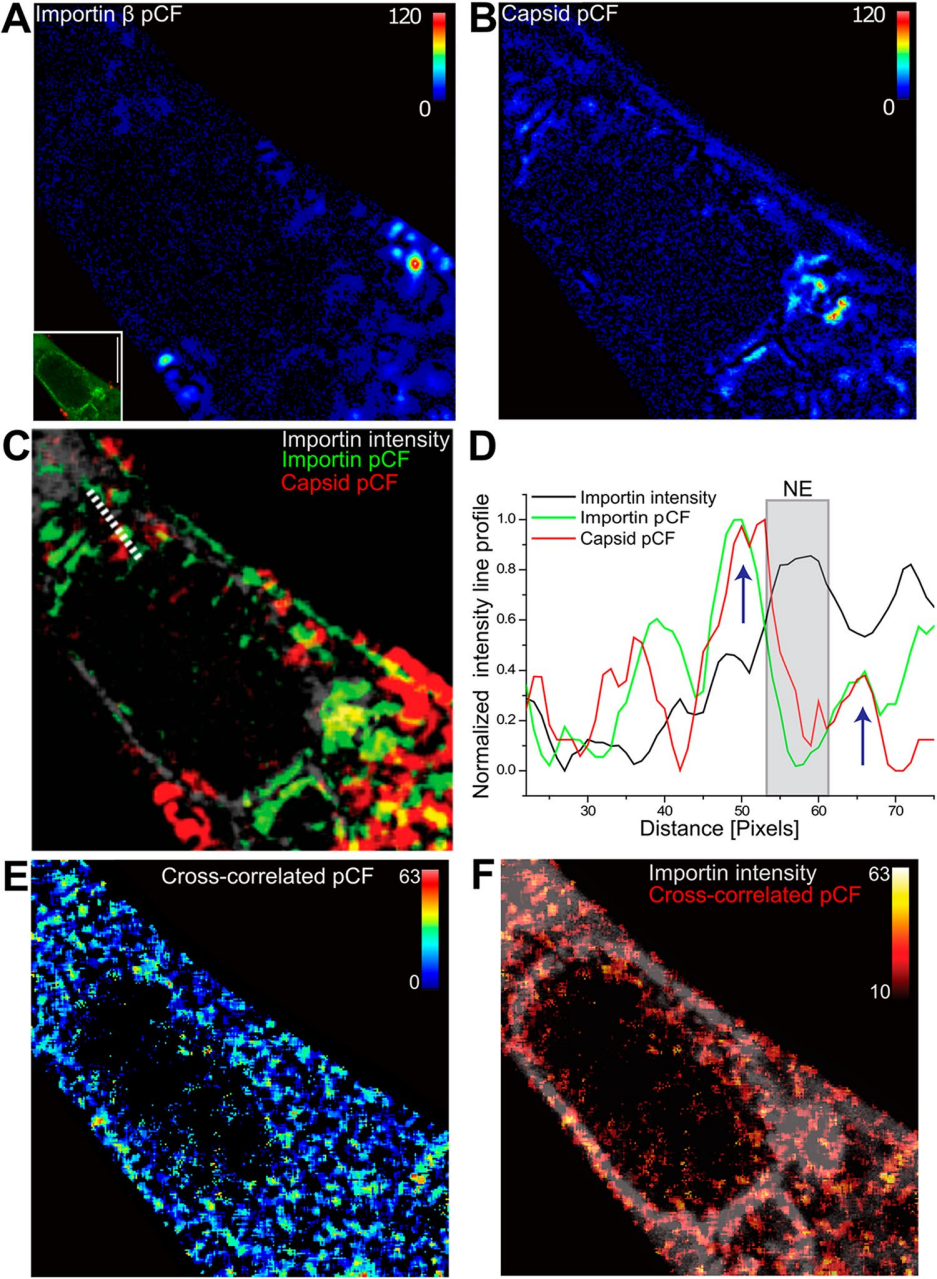
Image pair-correlation analyses of importin β and capsid movement. (**A**) Confocal microscopy image of a CPV-A594 (red) infected cell stably expressing importin β-GFP (green) at ~1 h p.i (lower left). The image pCF analysis of intracellular movement of importin β and capsids (**B**). The intensity corresponds to the correlation integral of G(r, r + 4). (**C**) Overlay of importin β pCF (green) and capsid pCF (red) with the importin intensity image shown in (A, the lower left corner). (**D**) Normalized intensity line profile analysis of importin β pCF and capsid pCF overlay as a function of distance (pixels) from the NE (gray zone) showing simultaneous movement of importin β and capsids across the NE. White dashed line shows the positive correlation of capsid and importin β movements from cytoplasm to nucleoplasm. (**E**) Cross-correlation of importin β and capsid pCFs. (**F**) The overlay of cross-correlated pCF with importin β intensity image (gray). Scale bar, 10 µm.

The transport of macromolecules across the NE is mediated by transport receptors of the importin β superfamily. Importin β recognizes its substrates in the cytoplasm and transports them through nuclear pores into the nucleus^38^. To study the mechanisms of virus translocation from cytoplasm into the nucleoplasm, we used pCF to visualize the movement trajectories of capsids and importin β across the NE at 1 h p.i. The analysis indicated capsid movement mostly in the cytoplasm, but also at the NE and in the nucleoplasm (Fig. 5). Moreover, both infrequent movement of capsids and abundant movement of importin β across the NE were detected (Fig. 5A and B). Overlay of the pCFs revealed that translocation of a small portion of capsids from the cytoplasm into the nucleus correlated with that of importin β (Fig. 5E and F). Finally, cross-correlated pCF analysis verified that the capsids and importin β moved simultaneously from the cytoplasm across the NE into the nucleus. In summary, our results indicate that both cytoplasmic transport of capsids towards the nucleus and their passage across the NE are mediated by importin β.

## Discussion

Although the basic principles of parvovirus cellular entry are known, the dynamics in particular at single viral capsid level remain unclear. For detailed analysis, we thus combined different advanced image analysis techniques and showed that the motility of capsids ranges from fast to slow in the cytoplasm, at the NE and in the nucleoplasm.

Our 3D particle analyses of the cytoplasmic zone showed that the stepwise cellular entry of CPV capsids was followed by their accumulation in close proximity to the nucleus, corresponding to endosomal transport of capsids to the perinuclear area. This interpretation is in agreement with our N&B analysis showing the presence of slowly moving particles in the cytoplasm, with their brightness increasing towards the nucleus. These results are in line with those of our earlier studies on the endocytic uptake of CPV, and data with other parvoviruses, including adeno-associated virus 2 (AAV2), which have demonstrated the presence of capsids both in small vesicles scattered around the cytoplasm, and in large vesicles in the perinuclear area^5,22,28,31,41,42^. Our results also agree with the generally accepted model of size-distribution of cargo-filled endosomes and their perinuclear accumulation: small endosomes with little cargo are found in the cell periphery, whereas large endosomes rich in cargo are located closer to the nucleus^43–46^. Once accumulated in the perinuclear area and released from microtubules, the large vesicles move very slowly or are nearly immobile (velocity <1 µm/s)^44–51^.

Our ACF and N&B analyses showed fast moving particles in the cytoplasm and close to the NE. Part of them represent endosomes with multiple capsids as indicated by their brightness reflecting the relative capsid content. The dimmer fast moving particles at the NE area likely correspond to cytoplasmic capsids after their endosomal escape. Although we cannot rule out that a small fraction of these capsids in the cytoplasm move by diffusion^52^, our ACF data indicate non-diffusive microtubule- and dynein-mediated movement of these capsids in the vicinity of the NE. In line with our results, earlier single particle tracking studies indicated both fast free (*D* = 1.3 μm^2^/s) and slow endosomal (*D* = 0.6 μm^2^/s) movement of AAV virus in the cytoplasm^53^. Moreover, high-speed cytoplasmic transport of AAV2 at a velocity of 3.5 µm/s along the microtubules, and of AAV9 at ~2.0 µm/s have been reported^5,54,55^. Furthermore, the NE zone contained slow particles, which were as bright as the cytoplasmic fast ones. This indicates that some of the capsids were actively transported across the NE, while the others slowed down or were arrested at the NE. Consistently with this observation, an average import time of of ~54 ms through the NE has been reported for a recombinant AAV^6^.

The intranuclear diffusion of capsids included both slow (*D* < 2 µm^2^/s) and fast (*D* >2 µm^2^/s) dynamics. We postulate the observed slower diffusion rates to correspond to very slow or nearly immobile capsids restricted by interaction with NPC or chromatin. The average diffusion coefficient for the nucleoplasmic capsids was ~2.88 µm^2^/s, which is in agreement with our earlier findings showing that the theoretical diffusion coefficient for entire CPV capsids was 3.0 µm^2^/s, and for CPV viral-like particles, 4.3 µm^2^/s^56^. The presence of importins on the capsid surface could lead to their increased diameter ranging between ~1.9 nm and 3.3 nm (importin α/β heterodimers), which would result in slower diffusion^57^. The detected slower diffusion in the nucleoplasm is in agreement with this. The increased diffusion with an average of D ≈ 11 µm^2^/s indicated the presence of faster-diffusing particles, suggesting the presence of disassembled capsids and/or capsid remnants. Although our data do not allow for pinpointing the subunits into which the capsids dissociated, the average fast diffusion (11 µm^2^/s) is in the same order with the earlier reported 11.9 µm^2^/s for the PAGFP-VP2 monomer^56^. For comparison, intranuclear diffusion rates of 8.2 µm^2^/s for the PAGFP-VP2 trimer^56^ and 37.5 µm^2^/s for free fluorophores in the nucleoplasm have been shown^58^. Altogether, our results suggest that the nuclear import is followed by capsid disassembly in the nucleoplasm. The corresponding mechanism of capsid disassembly and its exact intranuclear location remain to be determined.

Using pCF overlay, we were able to follow the transportation of capsids with transport receptor importin β in the cytoplasm and across the NE. The analysis demonstrated that in the cytoplasm the capsids were co-transported with importin β, however, this does not exclude the possible participation of importin α in the process. In parallel, ACF analysis of the NE confirmed the movement of capsids (*D* ≈ 3.53 µm^2^/s). Notably, this finding is consistent with the cytoplasmic diffusion of importin β fluctuating from slow (4 µm^2^/s) to fast (11 µm^2^/s) upon NLS-cargo binding^59^. The parvoviral capsid of <30 nm is small enough to pass through the NPC with a maximum diameter of ~39 nm^57^. Consistent with this, AFM analysis demonstrated the presence capsids on the nuclear side of the NPC I hour post cytoplasmic microinjection. However, receptor-mediated translocation is required for the passage of particles larger than 4*–*5 nm and 40 kDa^60^. This is in line with recent recombinant AAV2 studies showing importin β -assisted nuclear import of capsids^42^. Our pCF data, however showed CPV-importin β –complexes also deep inside the nucleus. This signifies that at least part of the capsids entered the nucleus without dissociating from importin β within the nuclear basket. This indicates possible defects in the functional integrity of the NPC/NE shown earlier for the rat parvovirus H1 and the minute virus of mice^61,62^.

**Figure 6:**
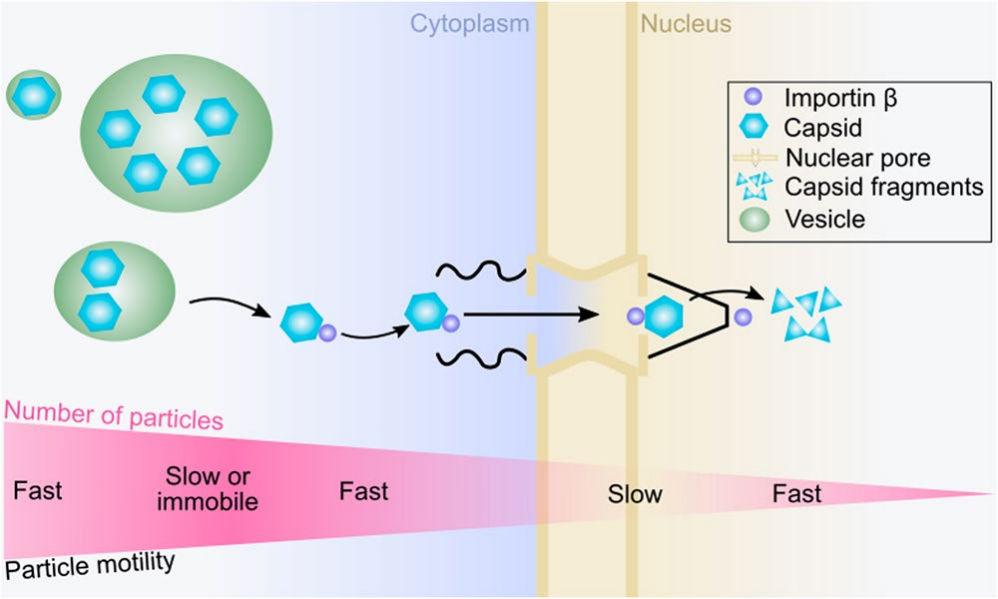
Schematic presentation of nuclear entry pathway of CPV. Upon release from the endosomal vesicles, CPV capsids are actively transported across the NE via importin β followed by disassembly in the nucleoplasm. However, the role of importin α in this process cannot be ruled out. During this transport, the motility of capsids fluctuates from cytoplasmic slow and fast, to nucleoplasmic slow and fast. The number of viral particles (purple gradient) is high distant from the nucleus and low near the NE. In the nucleus, higher amount of small particles is present suggesting for nucleoplasmic disassembly of the imported capsids.

To conclude, we applied image correlation analyses to quantify parvovirus capsid motion in the cytoplasm, NE and nucleoplasm at the single particle resolution unobstructed by particle concentration or intensity-induced technical limitations. With increased spatio-temporal resolution with regard to classical fluorescence imaging, we discovered how parvovirus capsids accomplish nuclear penetration (Fig. 6). Implementing quantitative image analysis methods and using pCF image correlation, we revealed for the first time the correlated movement of parvovirus capsids and importin β in the cytoplasm and across the NE in living cells. Our results contribute to comprehensive understanding of the dynamics of parvoviral entry, and open the field for further investigation of the complex virus-nucleus interactions.

## Materials and methods

### Cells, viruses and antibodies

Norden laboratory feline kidney (NLFK; Quality Control of Pfizer Animal Health, Lincoln, NE) cells were cultured in Dulbecco’s modified Eagle medium (DMEM, GlutaMAX^TM^) supplemented with 10% fetal bovine serum (FBS), 1% non-essential amino acids, and 1% penicillin-streptomycin (Gibco, Paisley, UK) at 37 °C in a presence of 5% CO_2_. NLFK cell lines stably expressing lamin C-EGFP (a generous gift from Juliet Ellis, University College London, UK) and transient expression of importin β-GFP (a generous gift from Patricia Lavia, Institute of Molecular Biology and Pathology CNR, Roma, Italy) was established by transfection at a confluence of 70% using TransIT®-2020 transfection reagent (Mirus Bio LLC, Madison, WI) according to the manufacturer’s instructions.

CPV type 2 capsids were derived from an infectious plasmid clone p265 by transfection of NLFK cells and the viruses were grown, isolated and concentrated as previously described^63,64^. For live cell imaging, the capsids were conjugated with Alexa 594 (CPV-A594, ratio of ~6–8 fluorophores per particle) and for 3D particle segmentation with Alexa 488 (CPV-A488) (ratio of ~6–8 fluorophores per particle, 2 µg of capsids/ml). For live cell imaging the cells were inoculated with virus and incubated at 37 °C for 45 min in a humidified incubator (5% CO_2_). Next, cells were washed and stabilized for 15 min on 37 °C preheated motorized microscopy stage. Cells were imaged at the heated stage for the duration of the infection.

For 3D particle segmentation analysis, α-microtubules were detected with a rabbit monoclonal Ab (EP1332Y, ab52866, Abcam, Cambridge, UK) followed by goat anti-rabbit Alexa 633-conjugated secondary Ab (Thermo Fisher Scientific, Waltham, Massachusetts, USA).

### Confocal microscopy of fixed cells

For laser scanning confocal microscopy, cells were grown on glass cover slips, fixed at set time intervals with 4% PFA and permeabilized with 0.1% Triton X-100 in PBS supplemented with 1% BSA and 0.01% sodium azide. The cells were embedded with ProLong Gold antifade reagent with 4′, 6-diamidino-2-phenylindole (DAPI) (Molecular Probes, Eugene OR). The images were acquired with Olympus FV-1000 confocal microscope with a UPLSAPO 60× oil immersion objective (NA = 1.35). For 3D particle segmentation analysis, images of size 640 by 640 pixels were acquired with line averaging of 4 with voxel size 55 nm in the x and y, and 150 nm in the z dimension (zoom factor 6). Alexa 488 was excited with a 488-nm argon laser, and fluorescence was collected with a 510- to 540-nm band-pass filter, and Alexa 633 was excited with a 633-nm He-Ne laser, and the fluorescence was collected with a 647-nm long-pass filter. DAPI was excited by a 405-nm diode laser and monitored with a band-pass filter of 460 to 500 nm. Deconvolution of acquired images was performed with Huygens Essential software (SVI, Netherlands) and using signal-to-noise ratio of 7.

### 3D particle segmentation analysis

To determine the average volume, total intensity, and distributions of the fluorescent capsid -containing particles as a function of the distance from the NE (n = 20 cells), a 3D watershed 3D segmentation algorithm was used^65^. The volume, intensity and location of each particle were analysed using 3D Objects Counter function^66^ in Fiji software^67^. For every object, the distance of its center of mass from the NE was determined (closest distance to the edge of nucleus detected with DAPI). The distance values from the NE were then divided to 500 µm-wide bins and the volume and intensity of the objects were averaged over every bin.

### Fluorescence correlation and fluctuation analyses

To analyze the transit of viral particles from the cytoplasm to the nucleus, confocal microscope line scans were performed across the nuclear lamina of NLFK cells stably expressing lamin C-EGFP (green) and infected with fluorescently labeled capsids (CPV-Alexa594, red) at ≤2 h p.i from three replicates. An Olympus FV1000 microscope was used with a 60× water immersion objective with a numerical aperture (NA) =1.2. A pixel time of 2 µs/pixel for 32,000 lines was used to obtain the pixel-based fluorescence intensity maps for lamin C-EGFP and CPV-A594. The pixel size was 41 nm in x/y, and the total time per experiment was ~48 s.

Viral diffusion coefficients were determined from the carpet ACF analysis. ACF was used to characterize the fluctuations of fluorescence intensity in a pixel over time in space to visualize the transition time of the particle. Carpet analysis, ACF and pCF calculations were carried out using SimFCS (Laboratory of Fluorescence Dynamics) and MATLAB (The Mathworks Inc., 2010). The line scan ACF data for each pixel was fitted using a Matlab routine to single component FCS equation to find an average apparent diffusion and number of particles. This diffusion data was subcategorized into two divisions with the slow particles entailing virus movement in endosomes or in the nucleus and the fast particles encompassing single virus capsids and/or capsid fragments and dissociated fluorophores. This categorization was done based on the known velocities of dynein motors, endosomal vesicles and cytoplasmic viruses^5,68–71^.

For line pCF analysis, two pixels located ~20 pixels (20 × 40 nm = 800 nm) away were correlated and the pair correlation carpet was drawn. The pCF was used to show the diffusion translocation trajectories of capsids and importin β along the scanned line measured by the temporal cross correlation of the two objects at a given distance. The pair correlation function was defined for two pixels at a distance *d* and time delay *τ* as

1 $$pCF(\tau ,d)=\frac{ < F(t,0)\,F(t+\tau ,d) > }{ < F(t,0) >  < F(t,d) > }-1$$

where *F*(*t*, *r*) is the fluorescence intensity at position *r* at time *t*^16^.

N&B analysis was used to derive the number and brightness of viral capsids from laser scanned lines^15^. The variables were defined as apparent number of molecules, *N* = <*k*>^2^/*σ*^2^ and apparent molecule brightness, *B* = <*k*>/*N* where <*k*> is the average of the intensity of time and *σ*^2^is the variance of intensity. Number and Brightness analysis was done for two data sets.

We segmented 30000 lines into a) (1 line) × 300 lines × 100 dataset categorized as fast, b) (100 line average) × 100 lines × 1 dataset categorized as slow. This slow category matches the slow category used in ACF. As tabulated under Supplementary Table S1, the number and brightness data is classified under the diffusion coefficient reported for that pixel.

pCF image analyses were carried out in SimFCS program (Laboratory of Fluorescence Dynamics). The pCF was used to show the diffusion translocation trajectories of capsids and importin β. For this, we used two methods: (1) pCF amplitude colocalization to identify trajectory of CPV and importin separately and to visualize the colocalized trajectory. The pCFs were calculated separately for a pixel distance (d) of 800 nm (d = 4 pixels) and overlaid to identify co-localized trajectories. (2) Cross-correlated pCF amplitude to identify the pair correlation of co-transported molecules. The cross-correlated pCF was calculated for capsid and importin movements and the pCF analysis was performed on the cross-correlated channel showing the probability of a co-transport for two species at a distance of 800 nm at the same time.

## Supporting Information Methods

### Atomic force microscopy

To introduce viral capsids to the NE, capsids were microinjected into the cytosol of *Xenopus laevis* oocytes. At 60 min post injection the nuclei were isolated and the NE was spread on mica either the cytoplasmic or nucleoplasmic side facing up^72^. The application of AFM to structural investigations of the virus capsids and NE has been described in detail in^73^. Veeco Multimode atomic force microscope equipped with a nanoscope V controller was applied with OTR4 AFM tips (Olympus, Tokyo, Japan). The images were recorded in tapping mode (cantilever spring constant 0.04 N/m), with 512 lines per screen, at a scan rate of 1.5 Hz.

### Data availability statement

All data are available on request from the authors.

## Electronic supplementary material

Supplementary information
