# Supplementary material for: Viral highway to nucleus exposed by image correlation analyses
Source: Sci Rep. 2018 Jan 18;8:1152. doi: 10.1038/s41598-018-19582-w (PMC5773500; doi:10.1038/s41598-018-19582-w)
Supplement: Supplementary file 1 — Supplementary information [file 41598_2018_19582_MOESM1_ESM.pdf]

Viral highway to nucleus exposed by image correlation analyses

Elina Mäntylä, Jenu V Chacko, Vesa Aho, Colin R Parrish, Victor Shahin, Michael Kann, Michelle A Digman, Enrico Gratton & MaijaVihinen-Ranta

Supplementary information

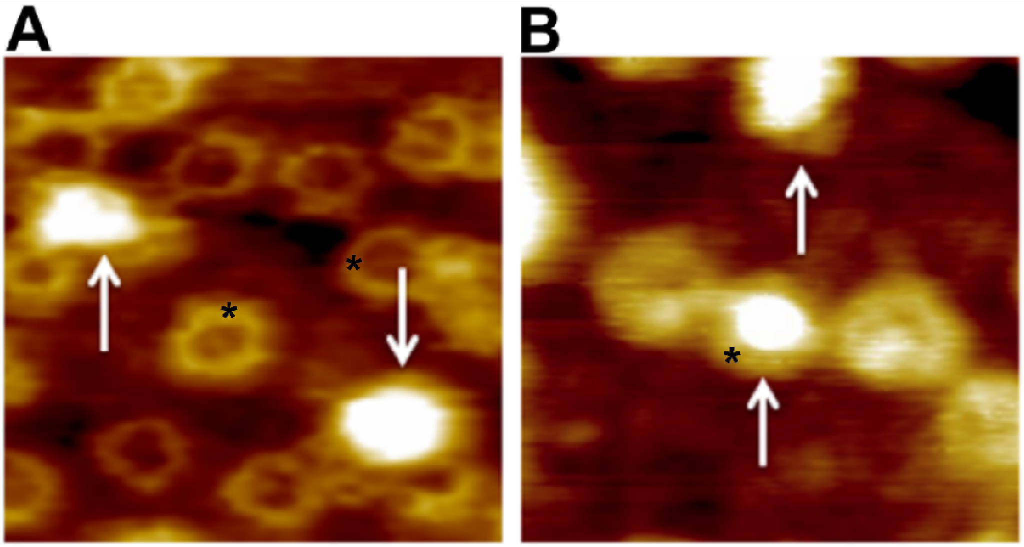

S1 Fig. Capsid interaction with NPCs. Atomic force microscopy (AFM) height images, which show capsids (A) on the cytoplasmic and (B) nucleoplasmic side of nuclear envelope (NE) at 1 h post injection of capsids into the cytosol of *Xenopus laevis* oocytes. The arrows show capsids located directly on top of the nuclear pore complexes (NPCs) marked with an asterisk. Image width is 0.6 µm.

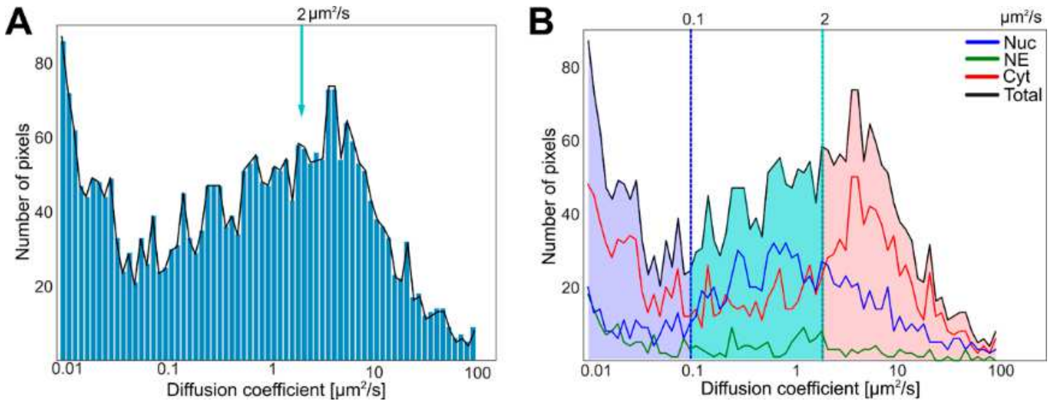

S2 Fig. Intranuclear capsid diffusion. (A) Autocorrelation function (ACF) analysis of intracellular capsid movement. (B) Histogram of apparent diffusion coefficients measured by fitting ACF carpets coefficients describing capsid movement in the cytoplasm, at the NE and in the nucleoplasm. Total capsid diffusion (black) and diffusion cutoffs at 0.1 and 2 µm²/s are indicated.

S1 Table. Intracellular diffusion, number and brightness of capsids.

Diffusion [µm²/s]	Zone	D_{app}	Number (1/G0)	Number fast [A.U.]	Number slow [A.U.]	Brightness fast [ε]	Brightness slow [ε]
<0.1	Cyt	0.02±0	0.02±0		0.34±0.03		1.79±0.48
	NE	0.01±0	0.03±0.01		0.29±0.03		0.83±0.31
	Nuc	0.02±0	0.01±0		0.35±0.03		0.54±0.14
0.1-2.0	Cyt	0.72±0.07	0.02±0		0.26±0.02		0.52±0.05
	NE	0.75±0.07	0.03±0.01		0.29±0.02		0.35±0.02
	Nuc	0.67±0.03	0.01±0		0.35±0.03		0.3±0.01
>2	Cyt	14.58±3.22	0.04±0.01	20.1±1.82		0.27±0.01	
	NE	12.48±1.98	0.07±0.01	25.5±2.38		0.26±0.01	
	Nuc	13.41±1.26	0.03±0	32.99±2.68		0.26±0	

Number and brightness (N&B) analysis of the capsid movement in the cytoplasm (Cyt), at the NE and in the nucleoplasm (Nuc). Viral particles were categorized into three classes of mobility: very slow or immobile (<0.1 µm²/s), slow (0.1-2 µm²/s), and fast (>2 µm²/s), based on their ACF-derived apparent diffusion (D_{app}). The total number of particles (1/G0), the number [A.U.] and the molecular brightness [ε] of particles in each class are shown.
